# Book Reviews

**Published:** 1918-08

**Authors:** 


### BOOK REVIEWS

Books mentioned may be inspected at and ordered through this office. So far as possible, books received in any month will be reviewed in the issue of the second month following. Pamphlets, quarterly and similar periodicals, reports, transactions, etc., will, as a rule, merely be mentioned.

**The Treatment of War Wounds.** By W. W. Keen, M. D., L.L.D., Emeritus Professor of Surgery, Jefferson Medical College, Philadelphia. Second Edition, Reset. 12mo 276 pages, illustrated. Philadelphia and London: W. B. Saunders Company, 1918. Cloth, $2.00 net.

Within about six months after the appearance of the first edition, a new edition, largely rewritten, has been called for. Major Keen has filled his book with facts obtained by communication with surgeons engaged in actual war work. The Carrel-Dakin method is given in the minutest detail, as well as acriflavine, mercurophen, eupad, eusol, dichloramin-T, tetanus and gas gangrene prophylaxis, the use and preparation of No. 7 Paraffin for burns, the localization and removal of foreign bodies by X-rays, and the treatment of wounds in various locations. A number of personal letters from experts in the service add much to the interest and value of this most timely volume.

---

**The Ungeared Mind.** By Robert Howland Chase, A. M., M. D., Physician-in-Chief Friends Hospital (for Mental Diseases). Cloth. Price, $2.75 net. Pp. 351, with 6 illustrations. Philadelphia: F. A. Davis Company, 1918.

Under this comprehensive caption the author has gathered together a few chapters comprising ''the observations and experiences of a lifetime.'' He writes for the general reader as well as for the physician who is interested particularly in psychology and its problems. With a genial outlook on the seamy side of life and a whimsical sympathy with minds that are out of gear, he has given us a mass of material unusual in any book and calculated to entertain as well as instruct the reader. The book is not a manual of a textbook, but he who reads it for knowledge will find unexpected supplies,—bits of wisdom not announced on the title page or noted in the index.

---

**Emergencies of a General Practice**, by Nathan Clark Morse, A. B., M. D., F. A. C. S., Surgeon to Emergency Hospital,

Eldora, Iowa; District Surgeon Chicago Northwestern Railway, Minneapolis & St. Louis Railway; Ex-President Iowa State Association of Railway Surgeons, etc. Author of "Post-Operative Treatment." Cloth, 8vo, 449 pages, 251 illustrations. St. Louis, C. V. Mosby Co., 1918. Price, $4.50.

This book covers in a most practical manner about all the emergencies likely to confront the general practitioner. Foreign bodies, asphyxiation, surgical and medical emergencies, food poisoning, burns, thermic fever, convulsions, bites, stings, retention of urine, etc., are all dealt with in a ready way. There is a chapter on obstetrical emergencies, and nearly a hundred pages on the treatment of poisoning. Fractures, dislocations and amputations are considered from an emergency standpoint. The value of the book is enhanced by the author's rich experience in dealing with the conditions that he describes.

---

**1917 Collected Papers of the Mayo Clinic,** Rochester, Minn. Octavo of 866 pages, 331 illustrations. Philadelphia and London: W. B. Saunders Company, 1918. Cloth, $6.50 net.

This is Volume IX of the Mayo Clinics. It presents 81 monographs on a wide range of topics—from the transplantation of whole organs to the war's influence on medicine. A fair balance is maintained between medical and surgical articles, and there are several papers of especial interest to the laboratory expert. The reviewer feels rewarded by a perusal of the paper by the Editor of this volume, Mrs. M. H. Mellish, on "Medical Journals from the Standpoint of the Contributor." If its spirit could be carried into practice it would bring about a much better understanding between the contributor and the editor who publishes his contribution; and the readers of medical journals would benefit by the result.

---

**The Essentials of Materia Medica and Therapeutics for Nurses,** by John Foote, M. D., Assistant Professor of Therapeutics and Materia Medica, Georgetown University School of Medicine; Instructor in Materia Medica and Therapeutics, Providence Hospital Training School for Nurses. Third edition, revised, enlarged and reset. Cloth, 310 pages, price, $1.75. Philadelphia: J. B. Lippincott Co., 1918.

This edition conforms to the Ninth Revision of the U. S. Pharmacopoeia, and gives also the official name of each drug recognized in the European pharmacopoeias. It is up to date, contains such information as nurses need concerning the newer preparations used in European military surgery, and will prove most useful to nurses as a textbook and a book of reference.

---

**Cancer: Its Nature, Causes, Diagnosis and Treatment.** By Robert Holmes Greene, A. M., M. D., F. A. C. S., Emeritus Professor of Surgery, Medical Department of Fordham University. Cloth, 172 pages, price, $1.50. New York: James T. Dougherty, 1918.

In preparing this little book the author has had the co-operation of John A. Killian, Ph. D., Pathological Chemist at the House of Calvary for Cancer. The cause and nature of cancer are briefly discussed. Under the head of diagnosis, the authors present their methods of determining the total sulphur and the neutral sulphur in the urine. This determination is of great diagnostic significance to them because of the fact that an increase of the percentage of neutral sulphur in the urine is found to exist in the presence of cancer, as a general rule, while the total amount of sulphur excreted is diminished. It is evident that carcinoma disturbs the normal balance between the sulphur components. This fact is very useful in making an early diagnosis of cancer. In the treatment of cancer, early operation is recommended in suitable cases. Where medicinal measures are indicated, the author favors the use of oxidizing agents like Selenium and Tellurium. The book is written in a spirit of fairness and the personal views of the authors are put forth modestly, without extravagant claims. There should be an index.

---

**Interpretation of Dental and Maxillary Roentgenograms,** by Robert H. Ivy, M. D., D. D. S., Major, Medical Reserve Corps, United States Army; Associate Surgeon, Columbia Hospital, Milwaukee; formerly Instructor in Oral Surgery, University of Pennsylvania. Cloth, 146 pages, 259 illustrations. Price, $2.50. St. Louis: C. V. Mosby Company, 1918.

The author believes that it is easier to make a good roentgenogram than to interpret it, and he devotes his book to the latter part of the specialty and limits his field to the teeth and jaws. There are two excellent chapters on the anatomy and pathology of these regions, after which two-thirds of the book is given to the interpretation of roentgenographic findings about the teeth and jaws, as illustrated in over 200 excellent reproductions of films and plates. The volume is a model of book making and a boon to the student of this essential factor in the diagnosis of dental conditions.

---

**Oral Sepsis in Its Relationship to Systemic Disease,** by William W. Duke, M. D., Ph. B., Kansas City, Mo., Professor of Experimental Medicine in the University of Kansas School of Medicine; Professor in the Department of Medicine in Western Dental College, etc. Cloth, 124 pages, 170 illustrations. Price, $2.50. St. Louis: C. V. Mosby Company, 1918.

The teachings of this book are based on the study of more than one thousand medical cases from whom over eight thousand dental films were taken. The author believes, as a result of these consultations and studies, "that the teeth should be taken into account in nearly every medical case." He writes clearly and convincingly on pyorrhea alveolaris, alveolar abscesses, metastatic infection, toxic effect or oral sepsis, and the influence of oral sepsis on nonrelated infection, headache, and other conditions. The illustrations are photographed from roentgenograms—clearly made and clearly interpreted. A copious bibliography and index complete this most elegant and effective contribution to an important branch of medicine and dentistry.

---

**The Treatment of Cavernous and Plexiform Angiomata by the Injection of Boiling Water (Wyeth Method),** by Francis Reder, M. D., F. A. C. S., Visiting Surgeon to City Hospital; Consulting Surgeon to St. John's Hospital and Missouri Baptist Sanitarium, St. Louis. Cloth, 12mo, 75 pages, 26 illustrations. Price, $1.50. St. Louis: C. V. Mosby Company, 1918.

Dr. John A. Wyeth contributes a brief introduction to this brochure. The author discusses angiomata in some detail and describes the Wyeth method with the aid of photographs from his own cases. He says, "Of 104 cases subjected to this method, I have had no failures to record." The book is a sufficient guide to the technic of boiling water injections, and its utility is enhanced by an index.

---

**Progressive Medicine**—a quarterly digest, etc., edited by Hobart Amory Hare, M. D., assisted by Leighton F. Appleman, M. D. Vol. II, June, 1918. Paper, 376 pages, illustrated. Price, $6.00 per annum. Philadelphia: Lea & Febiger, 1918.

Contents: hernia; surgery of the abdomen, exclusive of hernia; gynecology; disorders of nutrition and metabolism; —diseases of the glands of internal secretion, diseases of the blood and spleen; ophthalmology.

